# An attention enhanced CNN ensemble for interpretable and accurate cotton leaf disease classification

**DOI:** 10.1038/s41598-025-34713-w

**Published:** 2026-01-10

**Authors:** Md. Ehsanul Haque, Md. Tamim Hasan Saykat, Md Al-Imran, Ahsan Habib Siam, Jia Uddin, Debasish Ghose

**Affiliations:** 1 Department of Computer Science and Engineering, East West University, Dhaka, 1212 Bangladesh; 2 AI and Big Data Department, Woosong University, Daejeon, 34606 Republic of Korea; 3 School of Economics, Innovation, and Technology, Kristiania University of Applied Sciences, Bergen, Norway

**Keywords:** Computational biology and bioinformatics, Mathematics and computing, Plant sciences

## Abstract

Precise and timely identification of cotton leaf diseases is essential for sustaining crop yield and quality, yet manual inspection remains time-consuming, labor-intensive, and prone to error. Existing automated approaches are limited by insufficient dataset diversity, inconsistent evaluation practices, limited use of explainable AI (XAI), and high computational cost. To address these challenges, we propose an attention-enhanced CNN ensemble, namely *CottonLeafNet*, which integrates lightweight convolutional neural networks for accurate cotton leaf disease classification across two publicly available datasets. CottonLeafNet achieves state-of-the-art performance, obtaining 98.33% accuracy, a macro F1-score of 0.9833, Cohen’s kappa of 0.9800, a mean PPV of 0.9838, and an NPV of 0.9967 on Dataset D1, with an inference time of 0.51 s per image. On Dataset D2, it reaches 99.43% accuracy, a macro F1-score of 0.9942, Cohen’s kappa of 0.9924, a mean PPV of 0.9943, and an NPV of 0.9981, with a 0.40 s inference time. Moreover, a unified eight-class dataset created by merging both datasets yields a test accuracy of 99.08%. Robustness analysis under artificially induced class imbalance further confirms the model’s stability, with consistently strong macro F1-scores. To evaluate the generalization capability of the proposed CottonLeafNet, we conducted cross-dataset experiments, and the results indicate that the model maintains moderate performance even when trained and tested on different datasets. Gradient-Weighted Class Activation Mapping (Grad-CAM) visualizations demonstrate that CottonLeafNet reliably attends to disease-relevant regions, enhancing interpretability. Finally, real-time feasibility is validated through a web-based deployment achieving $\approx$1 s inference per image. These results establish CottonLeafNet as an accurate, robust, interpretable, and computationally efficient solution for automated cotton leaf disease diagnosis.

## Introduction

Cotton is one of the most important cash crops globally, used for making cotton fiber which is used in textile production and greatly affects agricultural economies^1^. However, it is greatly vulnerable to several leaf diseases, such as bacterial blight, leaf curl and target spot, that are the most prevalent as well as severe diseases in optimization of cotton crops^2^. These diseases not only decrease the yield of cotton but also decrease the quality of fiber and have an adverse effect on farmers’ economic status, market supply and its sustainability in textile industries. In addition to the damage that they cause in cotton, leaf diseases are a significant problem on many crops worldwide and increasingly result in estimates of huge production losses and consequently their impact on food security^3^. Early detection and timely differentiation of the infected leaves are thus crucial, as precaution measures can be taken in a perfect time to prevent further spread of the disease, supporting plant health and promoting sustainable agriculture^4^. Correct identification of leaf diseases also supports the precision application of pesticides and fertilizers, while minimizing excessive use of chemicals and environmentally friendly agricultural management practice^5^.

**Fig. 1:**
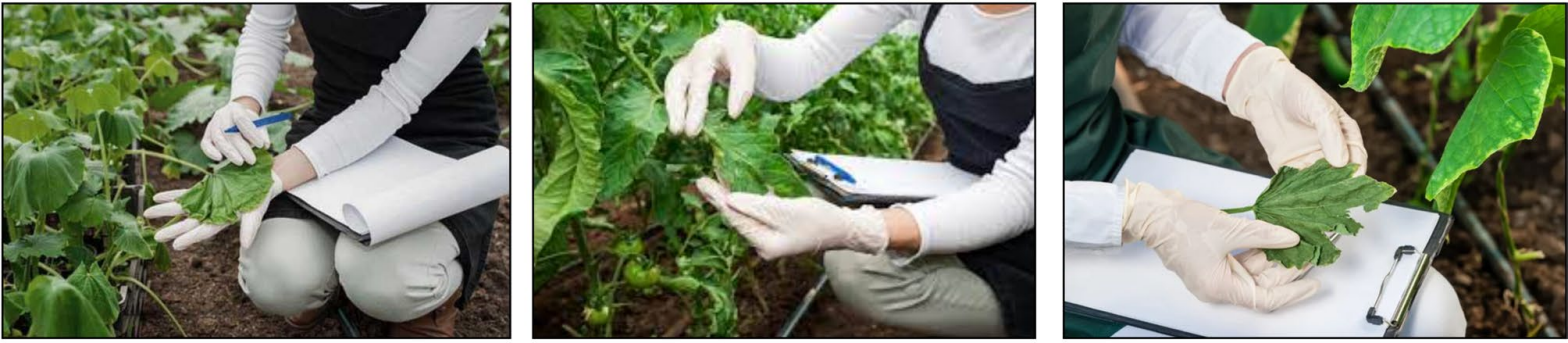
Manual inspection of leaves for disease diagnosis, illustrating the traditional approach of visually examining plants for symptoms. This process is labor intensive, time consuming, and prone to human error, especially in large scale agricultural fields^9^.

Conventionally, crop diagnosis is largely based on manual observations where agronomists or farmers use their eyes to observe whether a leaf had any disease symptom, discoloration, lesions, curling, or necrosis. This traditional method is very laborious, time consuming and subject to human error, especially in large scale fields where thousands of plants are to be tested within limited periods^6^. A typical manual inspection of the leaves for disease detection is shown in Fig. 1 where a human being observes the diseased leaves. The complexity of disease identification, fluctuating environmental conditions, along with overlapping or obscured leaves and early infection stages, further complicate the process of manual diagnosis^7^. These constraints highlight the urgent requirement for automated, reliable, and scalable disease detection systems to support a farmer in real time as well as on demand monitoring of the healthiness of crops^8^. Combining sophisticated imaging, machine learning and AI technologies, automatic leaf disease detection can also greatly enhance the speed, accuracy and repeatability of plant health assessment to support better disease control, greater yields and more sustainable agriculture.

Various papers have investigated the diagnosis of cotton leaf disease through machine learning and deep learning methods that show promising performance in the detection of various common diseases including bacterial blight, leaf curl, and target spot^10–12^. Nevertheless, these methods have significant shortfalls that prevent their effective implementation. Most of the current approaches are based on one dataset and therefore cannot be generalized to different environmental conditions and leaf variations. Besides, the computational cost is often ignored, and it is hard to implement models in resource-constrained agricultural environments. Overfitting is also a major problem, as models that are trained without adequate cross validation, or other statistical verification, tend to demonstrate unrealistic performance that does not work in practice. Also, model predictions are often not interpretable, which decreases their applicability in agriculture and agronomy. To deal with such challenges, there is the need to design models that are not only accurate but also computationally efficient, generalizable and interpretable.

To overcome these issues, we tested several attentive lightweight models with domain-specific preprocessing and light data augmentation to enhance the generalization of various leaf pictures. Owing to these models, we proposed *CottonLeafNet*, an attention-enhanced CNN ensemble that aggregates predictions from multiple CNN backbones using soft voting and incorporates XAI techniques to provide interpretable visualizations such as Grad-CAM. This framework is also supported by an interactive web application that is deployed to facilitate real time disease diagnosis where users can upload their leaf images and get instant predictions with confidence score and heatmap. To address overfitting and ensure dependable results, the model was evaluated using rigorous evaluation metric, including cross-validation and comprehensive statistical analysis. To assess the effectiveness of proposed framework, we used two publicly available cotton leaf datasets comprising of a combined set of eight unique disease categories, including common diseases like bacterial blight, target spot, leaf curl as well as Army worm damage. This wide range of data coverage guarantees that the suggested framework can differentiate between various types of diseases with high precision and be robust to changes in the appearance of the leaves, lighting, and the severity of the diseases. In general, the entirety of this methodology is that it is efficient in computational and interpretable aspects, thereby offering a viable, scalable, and high-performing method of automated cotton leaf disease detection in the real-life agricultural context.

We summarize the key contributions of this work as follows:

- We propose *CottonLeafNet*, an attention-enhanced CNN ensemble that integrates multiple lightweight CNN backbones with MHSA modules for efficient and accurate cotton leaf disease classification.
- We incorporate Grad-CAM–based explainability to ensure interpretable predictions by highlighting disease-relevant image regions.
- We conduct extensive evaluation on two public datasets (and their unified 8-class combination) using rigorous cross-validation, cross-dataset evaluation, and multiple reliability metrics to demonstrate robustness and generalization.
- We develop a practical, interactive web application enabling real-time inference with visual explanations, supporting field-level usability for farmers and agronomists.

Taken together, the novelty of our proposed framework lies in the integration of several lightweight attention models with XAI, enabling interpretable, reliable, and generalizable predictions, while the deployment of a real-time web application demonstrates its practical applicability, usability, and scalability in real-life agricultural scenarios.

### Literature review

Diseases of leaves are major problems in agriculture, which impact the health and productivity of crops^13^. To solve it, machine learning (ML) and transfer learning (TL) systems are actively used to solve the problem of automating the detection of leaves disease^14,15^. These methods facilitate the proper classification and detection of diseases using leaf images and eliminate the need to visually examine leaves by hand and enhance the scale of agricultural surveillance^16^.

In particular, cotton (Gossypium spp.) is very vulnerable to bacterial blight, leaf curl, and fungal infections that may decrease quality and yield of fibers dramatically^17^. There are critical roles of identifying these diseases early and classifying them properly in managing crops and ensuring sustainable farming^18^. In the past, a number of studies were devoted to the automation of cotton leaf disease diagnosis with the help of deep learning, and the section will review some of the most recent works, their findings, and limitations.

### Conventional CNN-based approaches

A deep learning model with a fine-tuned VGG16, VGG19, InceptionV3, and Xception models was recently proposed by Islam et al.^19^ to classify cotton leaf disease. Among them, Xception showed the best performance of 98.70% accuracy, which illustrates its better feature extraction ability. Moreover, an intelligent web application was created to enable realistic implementation in the farm environment. However, model interpretability, cross-validation, and robustness testing were not considered in the study, leaving much to be desired in explainability and overall performance validation.

Nazeer et al.^20^ developed a CNN-based system of cotton leaf curl disease detection and assigning its susceptibility to five levels with self collected and Kaggle data. It offered high accuracy at 99% performance over traditional R-CNN and SVM methods, with the help of a extensive preprocessing and augmentation. Nevertheless, there was no use of explainable AI methods, cross-validation and computational cost analysis which limits interpretability, strength, and feasibility of application.

Nagarjun et al.^21^ developed a deep learning model on cotton leaf diseases diagnosis with high precision through transfer learning models such as ResNet101, Inception v2, and DenseNet121. Nesterov accelerated gradient and image processing improved the accuracy of classification. The CNN had an accuracy of 99% and Inception v2 and DenseNet121 had 97.32 and 97.16% respectively. However, scientific rigor and practical applicability were restricted as cross-validation and explainable AI evaluation, computational analysis, and statistical significance testing were not performed.

Herkok and Ahmed^22^ came up with a transfer learning model to categorize seven cotton leaf diseases and healthy leaf by using a dataset of 6,158 images of various origin. VGG16 was the highest-testing model in the group of pre-trained CNNs with an accuracy of 95.02%. The evaluation based on classes indicated that there were issues caused by the inter class similarity especially between Target Spot and Bacterial Blight. Although the study functions well, the data used was relatively small, high intra class variability was observed and considerable problems might occur when generalizing the results to images in completely different field conditions.

Azath et al.^23^ propsoed a CNN-based model that identifies cotton leaf diseases and pests, such as bacterial blight, leaf miner, and spider mite, using 2,400 images with cross-validation in K-folds. The model has a total precision of 96.4% suggesting that it can be used in the field in real-time. The small size of the dataset, concentration on a specific four classes, and geographical data collection are the limitations that might make it challenging to generalize the results to other geographic areas or other types of diseases.

### Transformer-based and hybrid models

Similarly, Ahmad et al.^24^ introduced a deep learning model that combined Vision Transformers (ViT) and Swin Transformers with regular CNN models. The ViT model achieved 96.72% binary classification accuracy and 93.39% multiclass, which is higher than the traditional CNN and ResNet models when testing a dataset of 3,475 annotated images. Although the transformer-based method improved the representation of features, the research did not consider computational cost analysis, explainable AI, and validation on real fields, which limited the scale and interpretation of large-scale applications of agriculture.

Singh et al.^25^ proposed a hybrid architecture comprising the use of the BERT-based segmentation system, ResNet feature generation, and PSO optimization in cotton leaf disease detection, providing an accuracy of 98.5% on the PlantVillage dataset. Transformer-driven encoder was able to localize disease regions very well to enhance interpretability and strength over baseline CNN methods. However, the computational cost assessment, cross-validation consistency and explainable AI validation were not provided in the study, and the questions about generalizability and efficiency of practical implementation remain.

Rehman et al.^26^ proposed a hybrid model of cotton leaf disease monitoring, which consists of CNN, LSTM, and RNN and TLA+ formal verification of symptom requirements, assuring correctness in symptom requirements. Their proposed CNN achieved 98.7 and 98.6% accuracy and F1-score on a 3,601-image, six-class dataset, significantly outperforming the LSTM and baseline CNN models, and augmentation further increased the performance. Nevertheless, the study was conducted using a relatively small dataset, showed no XAI descriptions, and had high uncertainty with a large 95% confidence interval [0.286–0.380.286.380].

The proposed study by Aslam et al.^27^ introduced a synergistic deep learning model that combined the use of VGG16 and MobileNetV2 feature fusion with a StackNet ensemble of LSTM, SVM, and Random Forest classifiers in the detection of cotton leaf disease in seven classes. Augmentation with StyleGAN has eliminated the issue of class imbalance and has a 97% accuracy on Roboflow and PlantVillage combined datasets. Although it is generally applicable across datasets, the model is expensive to compute with 143M parameters and uses curated datasets, which can negatively affect scalability and real time agricultural application.

### Metaheuristic and optimization-based approaches

A metaheuristic optimized deep learning model that uses EfficientNetB3 and InceptionResNetV2 to classify cotton leaf disease was proposed by Gurjot Kaur et al.^28^. The method used SMOTE to balance the classes as well as hyperparameter maximization using GAs and interpretability through explainable AI (LIME and SHAP). The model had a high level of accuracy 98%,, showing a decent performance in six disease classes. However, the reliance of the dataset on the affected area and lighting conditions might restrain generalizability in other areas or initial infections.

### Advanced detection and attention-based approaches

The research by Hu et al.^29^ has proposed ACURS YOLO, a more powerful variant of the YOLOv11 that is designed to detect multi class cotton leaf diseases with cross domain adaptation that handles the issue of small target misses and background noise. The model combines U Netv2, which is an implementation of multi scale segmentation, CBAM attention, which is an implementation of disease focus, and C3k2_RCM in the neck, which is an implementation of long range context. Trained on 3,000 augmented images of six classes, ACURS YOLO got 94.6% mAP at 0.5 and 92.3% F1, which was better than the results of YOLOv11 and earlier versions. Ablation proved that U Net v2 with CBAM enhances recall by 8%.

RT-DETR-DFSA proposed by Mo and Wei^30^ is a lightweight detector of five cotton leaf diseases that handles the problem of small lesions, occlusions, and changing weather conditions. The model was able to achieve 87.14% and 84.96% accuracy before and after pruning, respectively, with 4.9M parameters by decoding 2D self attention into 1D dilated convolutions (DFSA) and using StyleGAN2 ADA with Fourier deblurring as data augmentation. Ablation experiments showed that DFSA and augmentation increased mAP by 5.91% and Grad CAM satisfies localization of the lesions, yet GAN generated images might create domain gaps in extreme situations.

Wang et al.^31^ came up with RF CottNet, a resource effective model, which uses the MobileViTv2 backbone to classify cotton disease and pests. With 4.9 M parameters, the model was found to reach 98.4% accuracy, 98.5% precision, and 3.8 ms inference time on the CCDPHD11 dataset. Even though it is very efficient, cross-validation and statistical significance tests were not conducted, and the validity on mild infections and real field conditions was not evaluated, which casts doubt on the strength and generalization.

Pinal Salot et al.^32^ proposed machine learning and deep learning pipeline in detecting early cotton leaf disease using images of rural fields in a four-class Kaggle dataset called salot2025cotton. This method included mean and median filtering, Gabor feature extraction and IDA based augmentation. Some of the classifiers such as KNN, Decision Tree, SVM, Random Forest and InceptionV3 gave the highest accuracy of 97%, which is higher than the traditional classifiers. Nevertheless, cross-validation, explainable AI, and the analysis of computational costs as well as statistical testing were not part of the study and the small sample size presents possible threats of bias and lack of generalization.

### Potential gaps and summary

Despite the strong performance reported in the reviewed studies, several major limitations remain unaddressed. First, most existing works rely on a single dataset, which restricts model applicability across diverse environmental conditions and agricultural practices^21,27^. Second, rigorous evaluation practices such as cross-validation, statistical significance testing, and robustness analysis are often omitted, which raises concerns about performance reproducibility and consistency^28,32^. Third, many approaches provide limited attention to explainable AI and model interpretability, although these aspects are important for supporting user trust in operational settings^20,31^. Finally, computational efficiency, including inference speed, memory footprint, and scalability, is not examined in sufficient detail. This is particularly relevant for transformer-based and ensemble architectures that require considerable computational resources and may be difficult to deploy in resource-constrained agricultural environments^26^.

Overall, these observations reflect recurring gaps in dataset diversity, evaluation rigor, interpretability considerations, and computational analysis across the existing literature on cotton leaf disease classification.

## Methodology

**Fig. 2:**
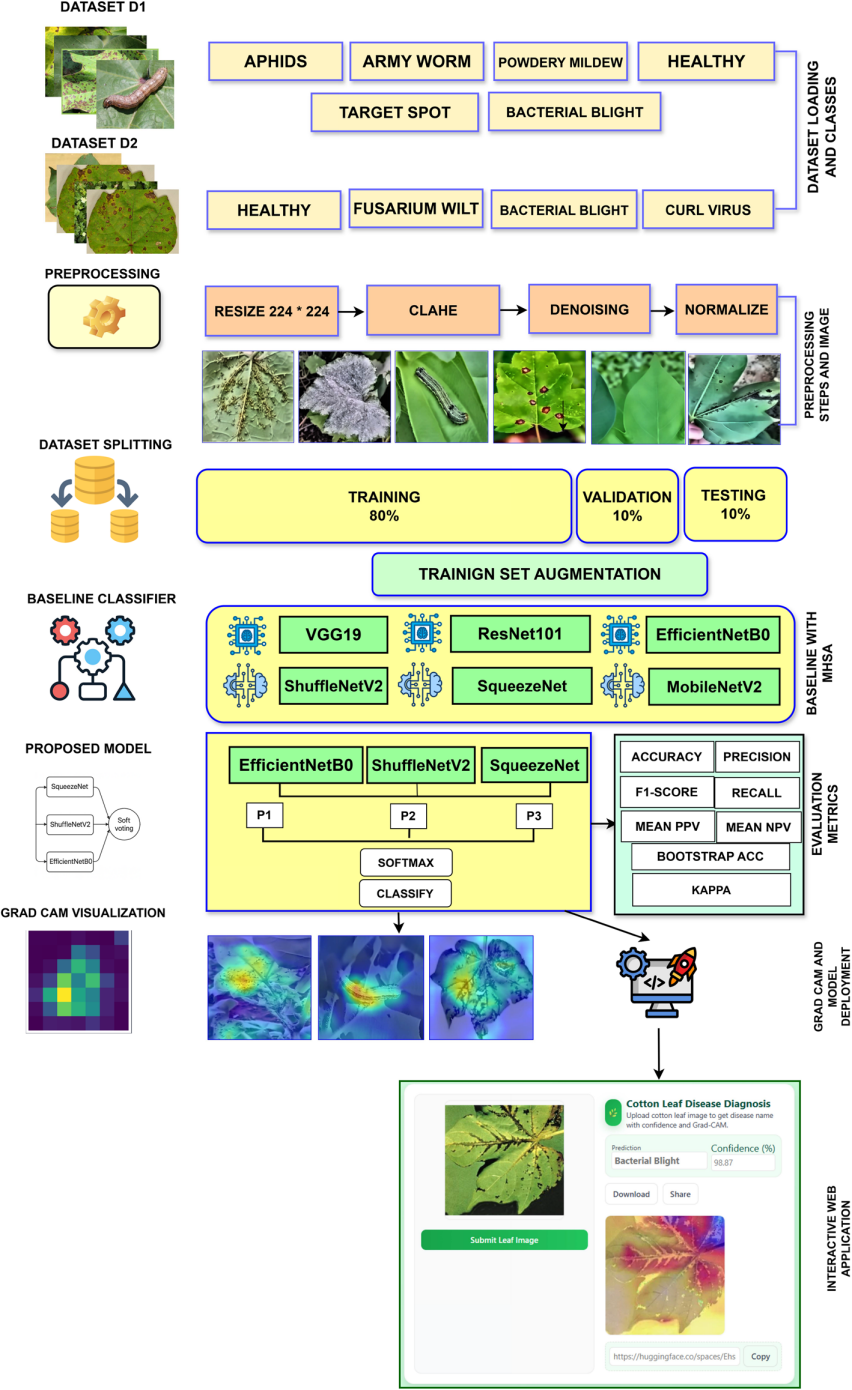
Workflow of the proposed CottonLeafNet framework showing the stages of data preprocessing, augmentation, model training, evaluation, explainability and deployment.

The overall workflow diagram of the proposed CottonLeafNet framework is presented in Fig. 2, showing all steps of data collection and preprocessing to model training, evaluation, explainability analysis and deployment. The figure gives a clear picture of how the datasets are transformed into actionable predictions using the proposed CottonLeafNet.

### Data acquisition

In this work, two publicly available Kaggle datasets based on cotton leaf disease classification were used denotes as D1 and D2^33,34^. Dataset D1 has 2,400 images divided into six disease classes, and Dataset D2 has 1,710 images that are divided into four classes. The reported 400 images per class in D1 are primarily the result of various types of augmentation applied to the original 40 images. The images in the two datasets were taken in both controlled conditions and in the real life settings, with variation in light, background and leaf position. Such variety will result in the overall representation of cotton leaf images and disease symptoms, thus making it possible to train, validate, and test the proposed classification models effectively. We also, merged both datasets to create a unified dataset comprising eight disease classes, enabling more comprehensive training and evaluation of the proposed framework. For the purpose of assessing generalization, a third publicly available Kaggle dataset, denoted as D3^35^, was included. Dataset D3 contains cotton leaf images grouped into seven disease categories and one healthy class, and it focuses exclusively on leaf-level symptoms. We have used the training part of the dataset here, it consists of a total of 6,628 images.

The per class distribution of images in each dataset is reported in Table 1. The table is structured to place D1, D2 and D3 side by side for easier comparison and readability.

**Table 1:** Class-wise distribution of images in D1, D2, and D3 datasets. The table highlights the number of images per class, providing an overview of dataset composition for controlled, real-life, and external conditions.

**Dataset D1**		**Dataset D2**		** Dataset D3 (Cross Dataset Evaluation)**	
**Class**	**Images**	**Class**	**Images**	**Class**	**Images**
Aphids	400	Bacterial Blight	448	Powdery Mildew	800
Army worm	400	Curl Virus	417	Cotton Boll Rot	960
Bacterial Blight	400	Fusarium Wilt	410	Army worm	800
Healthy	400	Healthy	425	Bacterial Blight	800
Powdery Mildew	400			Green Cotton Boll	880
Target Spot	400			Healthy	800
				Target Spot	788
				Aphids	800

**Fig. 3:**
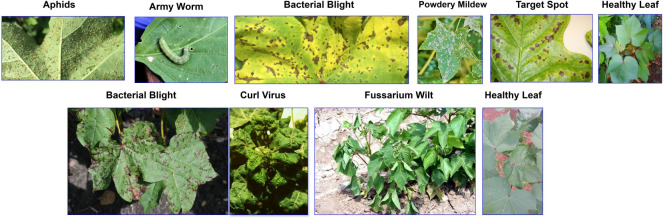
Representative images from the cotton leaf disease datasets. Top: Sample images of Dataset D1 that contain six disease classes. Bottom: Samples of Dataset D2 with four classes of diseases. The photographs were taken in the controlled set ups and in real life conditions.

Figure 3 shows some examples of both datasets where images of Dataset D1 are provided in the upper section and Dataset D2 on the bottom. This visualization offers an understanding of how different classes and environments have different leaf appearances and disease symptoms.

### Preprocessing and feature enhancement

All images were put through a sequence of preprocessing procedures to guarantee the homogeneity of input quality and to increase the characteristics involved in the classification of diseases. In the first step, the images were resized to the size of 224 $\times$ 224 pixels with area interpolation, and thus, input dimensions were harmonized without damaging crucial leaf features. Contrast Limited Adaptive Histogram Equalization (CLAHE) was next used on the Y channel (luminance) of the YUV color space with a clip limit of 2.0 and tile grid of $8 \times 8$ units. It is a step that enhanced local contrast without compromising chromatic data, which made the slight signs of disease like spots and lesions more noticeable. The images were post-processed with contrast enhancement, followed by non-local means denoising nature with parameters $h=10$, $hColor=10$, template window size=7, search window size=21 to eliminate camera and sensor noise but preserve fine structure information of the leaves. Finally, the images were clipped to the range 0–1 and back to 8 bit representation to ensure that the intensity levels of the images are consistent across the dataset and can be trained to produce a stable and efficient model.

**Fig. 4:**
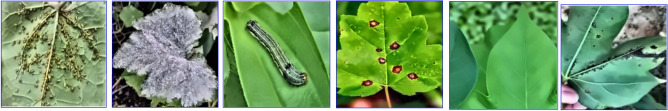
Representative preprocessed images after resizing, Y-channel contrast enhancement, denoising, and normalization. Each step improves feature visibility, reduces noise, and standardizes input for model training.

Figure 4 presents representative preprocessed images after all steps, demonstrating improved visual clarity and enhanced disease-relevant features suitable for downstream classification.

### Splitting dataset and augmentation

After preprocessing, the two datasets were divided into training, validation, and test subsets with the ratios of 80%, 10%, and 10% respectively. This division was to ensure that there were enough samples to train the models and at the same time have independent subsets to conduct a sound evaluation and hyperparameter optimization. In Dataset D1 with six classes, there were 1,920 images in the training set, 240 images in the validation set, and 240 images in the test set. In Dataset D2, which had four classes where variable original sizes were used, the training set comprised of 1,366 images, validation set consisted of 168 images and test set constituted 175 images. In addition, Dataset D3 was used exclusively as an external cross-dataset test set to evaluate the generalization capability of models trained on the combined D1 and D2 datasets. It contains the six classes common to the combined (D1+D2) dataset and serves as an independent benchmark for domain-shift evaluation.

Table 2 shows the distribution of images by class in each of the subsets of both datasets giving a complete picture of how datasets are composed. This split information is presented in detail, which assures transparency, promotes reproducibility and creates a clear base on which to base the model training, validation and performance assessment in the future.

**Table 2:** Class-wise distribution of images in training, validation, and test sets for Dataset D1 and D2, and cross-dataset validation set D3 (test only).

**Dataset D1**				**Dataset D2**				**Dataset D3 (Cross-Dataset Test)**	
**Class**	**Train**	**Val**	**Test**	**Class**	**Train**	**Val**	**Test**	**Class**	**Test**
Aphids	320	40	40	Bacterial Blight	358	44	46	Aphids	519
Army worm	320	40	40	Curl Virus	333	41	43	Army worm	560
Bacterial Blight	320	40	40	Fusarium Wilt	335	41	43	Bacterial Blight	485
Healthy	320	40	40	Healthy	340	42	43	Healthy	532
Powdery Mildew	320	40	40					Powdery Mildew	513
Target Spot	320	40	40					Target Spot	471

In order to make the training data even more varied, as well as increase the generalization of a model, a sequence of image augmentation methods was implemented to the training subsets. These augmentations consisted of random horizontal and vertical flips, rotations in a range of plus or minus 25 degrees, zooming with a factor between 0.8 and 1.2 times the original size and random changes to the brightness within a factor of 0.7 to 1.3. The original training images were tripled with a random mix of two augmentation functions, and thus, the actual amount of training samples was significantly increased without altering the underlying disease patterns.

**Fig. 5:**
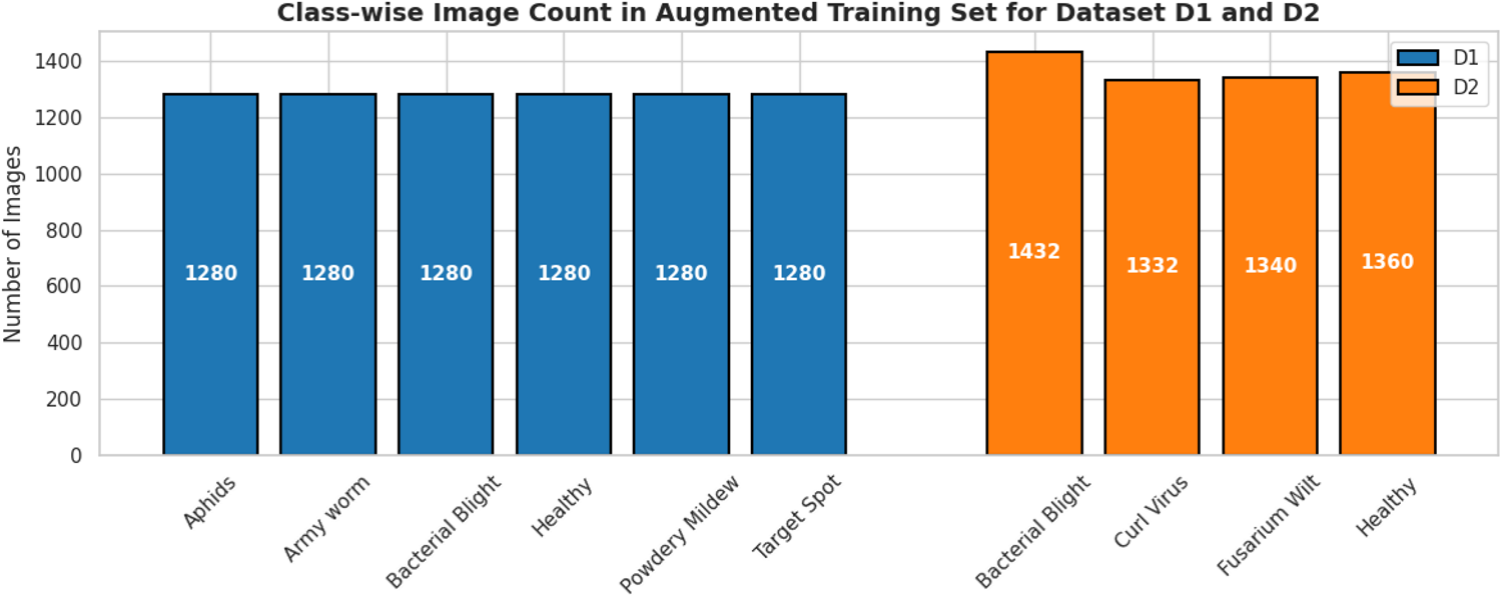
Class-wise distribution of images in the augmented training sets for both Dataset D1 and D2. Each original training image was augmented three times using random combinations of flips, rotations, zooms, and brightness adjustments, resulting in a substantial increase in training samples.

In Fig. 5, the total count of images of each class in the augmented training in both Dataset D1 and D2 is shown. The augmentation procedure proves effective in increasing variation in the training data, which makes the consequent classification models more resistant to variations in leaf looks, direction, and lighting.

### Baseline classifiers

To develop a strong foundation of classification of cotton leaf disease, we used six models of convolutional neural networks (CNNs), including ShuffleNetV2, SqueezeNet, Vgg19, ResNet101, MobileNetV2, and EfficientNetB0. The reasons why these models were selected are that they introduce a broad spectrum of design approaches and collectively present opposite views concerning the computational efficiency, feature representation, and predictive performance. In particular, ShuffleNetV2, 50 layer, and SqueezeNet, 18 layer, are small sized networks that can be deployed on low resource devices and can still be effective in processing images within a short amount of time but preserving important leaf features. By comparison, VGG19, 19 layers, and ResNet101, 101 layers, extract the deepest hierarchical feature that assists in identifying subtle variations in a leaf texture, lesion margins, and spot patterns. In the meantime, MobileNetV2, consisting of 53 layers, is also an efficient and representation balanced model based on inverted residual blocks and depthwise block separable convolutions. Lastly, with a total of 82 layers, EfficientNetB0 is a well performing model that is highly accurate, scaling the depth, width and resolution with a low computational cost. All these models enable a direct comparison of the predictive performance and efficiency in the cotton leaf disease recognition.

To enhance feature extraction and generalization we added MHSA in all models with four attention heads. This mechanism allows the networks to concentrate on the local specifics as well as bigger contextual trends that is imperative in the identification of subtle symptoms of the disease. We also inserted a dropout layer prior to the classification head in order to decrease overfitting. The last classifier will involve adaptive average pooling, flattening, dropout, and output probability of disease classes with a fully connected layer. Attention and dropout application also consistently provide every model with a better sensitivity and a reasonable comparison of performance. In general, this attention and dropout set of architectures offers a strict evaluation framework of baselines. It enables us to determine the impact of network depth, design selection and addition of features on classification accuracy, computational performance, and robustness of the detection of cotton leaf disease. This design allows lightweight and deeper networks to be compared and deployed in a variety of precision agriculture situations.

### Proposed CottonLeafNet: fused attention-based CNN for reliable cotton leaf disease classification

**Fig. 6:**
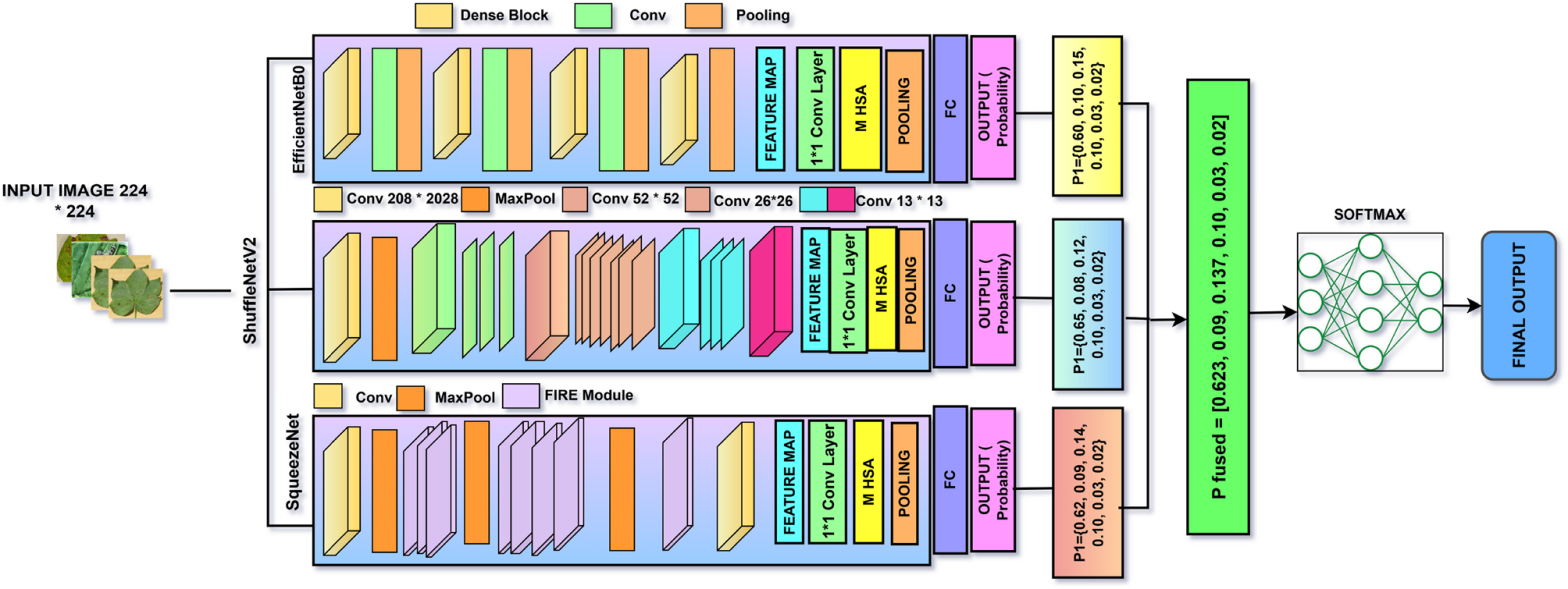
Architecture of the proposed CottonLeafNet model showing the backbone networks, prediction fusion strategy, and the integration of the soft voting ensemble for accurate cotton leaf disease classification.

The proposed *CottonLeafNet* framework leverages the top three highest performing baseline CNN models, enhanced with attention based feature integration, to accurately classify cotton leaf diseases. More specifically, SqueezeNet, ShuffleNetV2, and EfficientNetB0 serve as lightweight CNN backbone architectures that are each enhanced with a four-head MHSA module. MHSA module allows the network to pay attention to significant areas of the leaf images, both to capture local information, like lesions and spots, and to capture global information patterns. To reduce overfitting, a dropout layer is used prior to the classification head. The classifier is made of adaptive average pooling, flattening, dropout, and an adapted fully connected layer producing class probabilities. A soft voting ensemble is employed in order to build stronger predictions: given an input image, each model will use its backbone to extract feature maps, which are then refined by MHSA and a probability vector is produced by its classifier. The probability vectors across all three models are averaged and it is the model with the highest fused probability that is the final model that is predicted. The general workflow of CottonLeafNet is presented in Algorithm 1, which includes CottonLeafNet up to the final prediction, involving the examples of both Dataset D1 and Dataset D2 to show how probability vectors are combined and the final class is chosen. Also Fig. 6 shows the proposed CottonLeafNet architecture consists of the combination of several backbone networks and a soft voting ensemble in order to better feature representation and to increase the cotton leaf disease classification performance.

**Algorithm 1:**
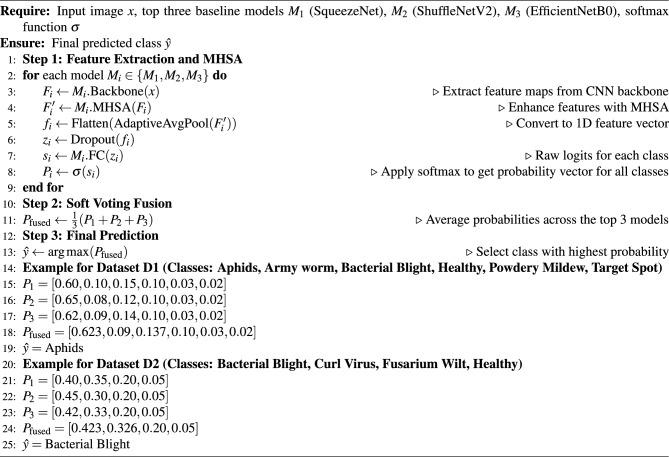
CottonLeafNet: Ensemble of Top 3 Baseline CNN Models with MHSA and Soft Voting

### Model training settings

The training settings and the hyperparameters used for both baseline CNN models and the *CottonLeafNet* are detailed in Table 3. Each model was trained on $224 \times 224$ input images with a batch size of 32 and optimized with Adam with a learning rate and weight decay of $1\times 10^{-4}$. The training was conducted for 50 epochs with early stopping 5 and a learning rate scheduler (ReduceLROnPlateau) to prevent overfitting and ensure stable convergence. In the case of CottonLeafNet, MHSA was used with 4 heads and a dropout rate of 0.3 to improve the feature representation. All baseline models, with the exception of the base learners of the CottonLeafNet model, had their backbone layers frozen. All of the experiments were conducted using an NVIDIA Tesla P100 GPU, which is efficient in terms of calculation and optimized performance between training.

**Table 3:** Training settings and hyperparameters used for CottonLeafNet and baseline models.

**Setting**	**Value/Description**
Models	ShuffleNetV2, SqueezeNet, VGG19, ResNet101, MobileNetV2, EfficientNetB0
Proposed Model	CottonLeafNet (Soft Voting)
Backbone Frozen	True (all models except CottonLeafNet base models)
Attention Heads	4
Attention Dropout	0.3
Batch Size	32
Image Size	224 $\times$ 224
Optimizer	Adam
Learning Rate (LR)	1e-4
Weight Decay (WD)	1e-4
Number of Epochs	50
Early Stopping Patience	5 epochs
Learning Rate Scheduler	ReduceLROnPlateau (monitor: val_loss, patience: 5)
GPU	NVIDIA Tesla P100 (Kaggle)

### Grad-CAM visualization for proposed CottonLeafNet

In order to improve the interpretability and develop trust in the proposed model predictions, we use Grad-CAM^36^ on it to see whether it focuses on disease-relevant areas or not. In CottonLeafNet, the Grad-CAM pixel importance is derived by computing the Grad-CAM for each individual base model in the ensemble and then averaging these maps to obtain an ensemble-level visualization. This approach highlights regions that are consistently important across all constituent models. This method of highlighting the most significant parts of cotton leaf images that affect the desired disease classification will enable the researcher and agronomists to comprehend and confirm that the architecture concentrates on the disease-specific features.

### Interactive web application for real-time prediction

In order to make it practically usable, we created an interactive web app with Python and Gradio on Hugging Face^37^ platform that enables users to upload cotton leaf images and receive a real-time prediction with the predicted disease type, confidence score, and Grad-CAM visualization to explain the prediction. This deployment will make it easily accessible to agronomists and farmers so that they can make quick decisions under field conditions.

## Results and discussion

This section presents the performance of *CottonLeafNet* on datasets D1 and D2, including classification results, ensemble effectiveness, and Grad-CAM visualizations to highlight disease-relevant regions. Key insights on accuracy, robustness, and interpretability are discussed.

### Testing performance of all evaluated models with MHSA attention

Table 4 presents the test performance of several CNN architectures on Dataset D1 and Dataset D2. In both datasets, CottonLeafNet performs significantly better than all of the baseline models in terms of precision, recall, macro-F1 score, and overall accuracy. In Dataset D1, CottonLeafNet reaches an accuracy of 98.33%, which is better than the next best model, EfficientNetB0, which only reaches 97.50%. Similarly, in Dataset D2, CottonLeafNet achieves an accuracy of 99.43% which shows that it is better at generalizing to a different dataset with different classes of diseases. The noteworthy improvements in macro-F1 and weighted-F1 scores across both datasets indicate that CottonLeafNet delivers consistently high performance across all classes, demonstrating its ability to accurately classify each disease category in the given balanced datasets. These results support the idea that a collection of the best performing models combining ideas of MHSA and soft voting can succeed to represent complementary features with more reliable and accurate predictions. The steady enhancements in both lightweight and deep networks indicate the effectiveness of the proposed ensemble architecture. Lastly, the evaluation metrics like ROC AUC and PR AUC, which are shown below the Table 5, further highlight the discriminative ability of the model and provide further information about its confidence and reliability on the class level, which offers a comprehensive picture of performance across all disease categories.

**Table 4:** Test performance comparison of CNN architectures on Dataset D1 and Dataset D2. The proposed CottonLeafNet achieves the highest classification accuracy, macro F1, and weighted F1 on both datasets, demonstrating superior generalization and robustness.

**Model**	**Dataset D1**				**Dataset D2**			
	**Precision**	**Recall**	**F1-score (Macro)**	**Accuracy (%)**	**Precision**	**Recall**	**F1-score (Macro)**	**Accuracy (%)**
ShuffleNetV2	0.9636	0.9625	0.9621	96.25	0.9889	0.9884	0.9886	98.86
SqueezeNet	0.9668	0.9667	0.9667	96.67	0.9838	0.9826	0.9829	98.29
VGG19	0.9548	0.9542	0.9542	95.42	0.9724	0.9709	0.9710	97.14
ResNet101	0.9608	0.9583	0.9585	95.83	0.9833	0.9826	0.9827	98.29
MobileNetV2	0.9643	0.9625	0.9627	96.25	0.9885	0.9884	0.9884	98.86
EfficientNetB0	0.9760	0.9750	0.9751	97.50	0.9885	0.9884	0.9884	98.86
**CottonLeafNet (Proposed)**	**0.9838**	**0.9833**	**0.9833**	**98.33**	**0.9943**	**0.9942**	**0.9942**	**99.43**

Table 5 presents class-wise ROC AUC and PR AUC for all models on Dataset D1 and D2. On Dataset D1, *CottonLeafNet* achieves perfect separation for Army worm and Bacterial Blight, while Aphids reaches 0.9999/0.9994. Healthy and Powdery Mildew maintain similarly high values, and Target Spot records 0.9975/0.9917, showing consistent sensitivity even for underrepresented classes. Baseline models exhibit slightly lower scores, reflecting variability in discriminative ability across disease types. In Dataset D2, *CottonLeafNet* attains 1.0 for Bacterial Blight, Curl Virus, and Healthy, while Fusarium Wilt reaches 0.9991/0.9972, demonstrating balanced performance across classes. Class-level comparison indicates that the ensemble approach enhances both precision and recall for visually subtle disease patterns.Below, we report Cohen’s Kappa, Brier score, and internal mean PPV and NPV to further quantify prediction reliability. These metrics provide additional insight into model calibration, uncertainty, and class-level performance, complementing the ROC AUC, PR AUC, and accuracy measures presented above.

**Table 5:** Class-wise ROC AUC and PR AUC of CNN architectures on Dataset D1 and Dataset D2. The proposed CottonLeafNet consistently outperforms baseline models across all disease classes in both datasets.

**Dataset**	**Class**	**ShuffleNetV2**	**SqueezeNet**	**VGG19**	**ResNet101**	**MobileNetV2**	**EfficientNetB0**	**CottonLeafNet**
		ROC/PR	ROC/PR	ROC/PR	ROC/PR	ROC/PR	ROC/PR	ROC/PR
**Dataset D1**	Aphids	0.9948/0.9839	0.9994/0.9972	0.9934/0.9779	0.9966/0.9881	0.9983/0.9930	0.9994/0.9972	**0.9999/0.9994**
	Army worm	0.9994/0.9969	0.9999/0.9994	0.9993/0.9965	0.9995/0.9976	0.9998/0.9988	0.9998/0.9988	**1.0000/1.0000**
	Bacterial Blight	0.9981/0.9915	0.9976/0.9882	0.9985/0.9930	1.0000/1.0000	0.9995/0.9975	0.9975/0.9884	**1.0000/1.0000**
	Healthy	1.0000/1.0000	1.0000/1.0000	0.9999/0.9994	0.9999/0.9994	0.9984/0.9936	1.0000/1.0000	**0.9998/0.9988**
	Powdery Mildew	1.0000/1.0000	0.9750/0.9750	0.9975/0.9903	0.9999/0.9994	0.9994/0.9971	1.0000/1.0000	**1.0000/1.0000**
	Target Spot	0.9946/0.9853	0.9974/0.9887	0.9984/0.9926	0.9956/0.9851	0.9940/0.9849	0.9980/0.9916	**0.9975/0.9917**
**Dataset D2**	Bacterial Blight	1.0000/1.0000	1.0000/1.0000	1.0000/1.0000	1.0000/1.0000	1.0000/1.0000	1.0000/1.0000	**1.0000/1.0000**
	Curl Virus	1.0000/1.0000	1.0000/1.0000	0.9988/0.9962	1.0000/1.0000	1.0000/1.0000	0.9991/0.9972	**1.0000/1.0000**
	Fusarium Wilt	0.9991/0.9972	1.0000/1.0000	1.0000/1.0000	0.9989/0.9968	0.9981/0.9934	1.0000/1.0000	**0.9991/0.9972**
	Healthy	0.9938/0.9896	0.9954/0.9912	0.9954/0.9912	0.9963/0.9924	0.9949/0.9906	0.9854/0.9847	**0.9986/0.9964**

### F1-score analysis on artificially imbalanced dataset

As our train-test-validation folds were balanced, the F1 scores reported in Table 4 do not fully capture the model’s performance under conditions of class imbalance. In order to create a more realistic situation and determine the resilience of the proposed model, we created an artificially imbalanced dataset by selecting a portion of samples in each category at random with keepratio = random.uniform(0.76, 1.0). Table 6 presents the F1-score–based performance analysis on an artificially imbalanced dataset. In both datasets, CottonLeafNet performs well, with the highest macro F1 score of 0.9857 on Dataset D1 and 0.9930 on Dataset D2, and weighed F1 score of 0.9858 and 0.9930, respectively. In contrast, other models have a little lower macro F1 score suggesting that they are a bit sensitive to class imbalance whereas their weighted F1 score and accuracy are relatively constant because of the effect of majority classes. Such findings further confirm that the Proposed CottonLeafNet provide consistent performance even if there is imbalance on the dataset which underlines its ability to be used in real-world conditions of agriculture.

**Table 6:** Comparison of Macro and Weighted F1 Scores of Different Models on Artificially Imbalanced Datasets D1 and D2.

Model	F1 Score (Macro)		F1 Score (Weighted)	
	D1	D2	D1	D2
ShuffleNetV2	0.9761	0.9930	0.9762	0.9930
SqueezeNet	0.9668	0.9723	0.9669	0.9718
VGG19	0.9275	0.9930	0.9288	0.9930
ResNet101	0.9761	0.9796	0.9761	0.9789
MobileNetV2	0.9767	0.9861	0.9765	0.9859
EfficientNet-B0	0.9759	0.9648	0.9766	0.9648
CottonLeafNet	0.9857	0.9930	0.9858	0.9930

### 5-fold cross-validation of all evaluated models

Table 7 shows the 5-fold cross-validation accuracy of all baselines models and the proposed *CottonLeafNet* ensemble on Datasets D1 and D2. In all the folds the models have consistent high performance with the mean accuracy of 99.56% on D1 with standard deviation of 0.0022 and 99.85% on D2 with standard deviation of 0.0009 which supports the overall better generalisation and stability of CottonLeafNet. The findings also show that deeper networks together with lightweight models have complementary advantages that effectively contribute to the overall performance of the ensemble.

**Table 7:** 5-Fold Cross-Validation Accuracy of All Models on Datasets D1 and D2.

**Dataset**	**Model**	**Fold 1**	**Fold 2**	**Fold 3**	**Fold 4**	**Fold 5**	**Mean** ± **SD**
D1	SqueezeNet	0.9798	0.9798	0.9714	0.9753	0.9701	0.9753 ± 0.0041
	VGG19	0.9740	0.9837	0.9805	0.9798	0.9772	0.9790 ± 0.0033
	ResNet101	0.9785	0.9876	0.9798	0.9818	0.9883	0.9832 ± 0.0040
	MobileNetV2	0.9824	0.9876	0.9857	0.9863	0.9850	0.9854 ± 0.0017
	EfficientNet-B0	0.9922	0.9839	0.9915	0.9883	0.9928	0.9908 ± 0.0018
	ShuffleNetV2	0.9902	0.9824	0.9785	0.9850	0.9772	0.9827 ± 0.0047
	CottonLeafNet	0.9974	0.9987	0.9928	0.9954	0.9935	0.9956 ± 0.0022
D2	SqueezeNet	0.9881	0.9844	0.9863	0.9909	0.9872	0.9874 ± 0.0021
	VGG19	0.9863	0.9918	0.9872	0.9863	0.9881	0.9879 ± 0.0020
	ResNet101	0.9918	0.9954	0.9909	0.9927	0.9945	0.9930 ± 0.0017
	MobileNetV2	0.9945	0.9954	0.9936	0.9909	0.9936	0.9940 ± 0.0017
	EfficientNet-B0	0.9945	0.9936	0.9954	0.9927	0.9945	0.9945 ± 0.0013
	ShuffleNetV2	0.9918	0.9881	0.9899	0.9927	0.9963	0.9918 ± 0.0028
	CottonLeafNet	1.0000	0.9973	0.9982	0.9982	0.9991	0.9985 ± 0.0009

### Proposed model misclassification analysis

**Fig. 7:**
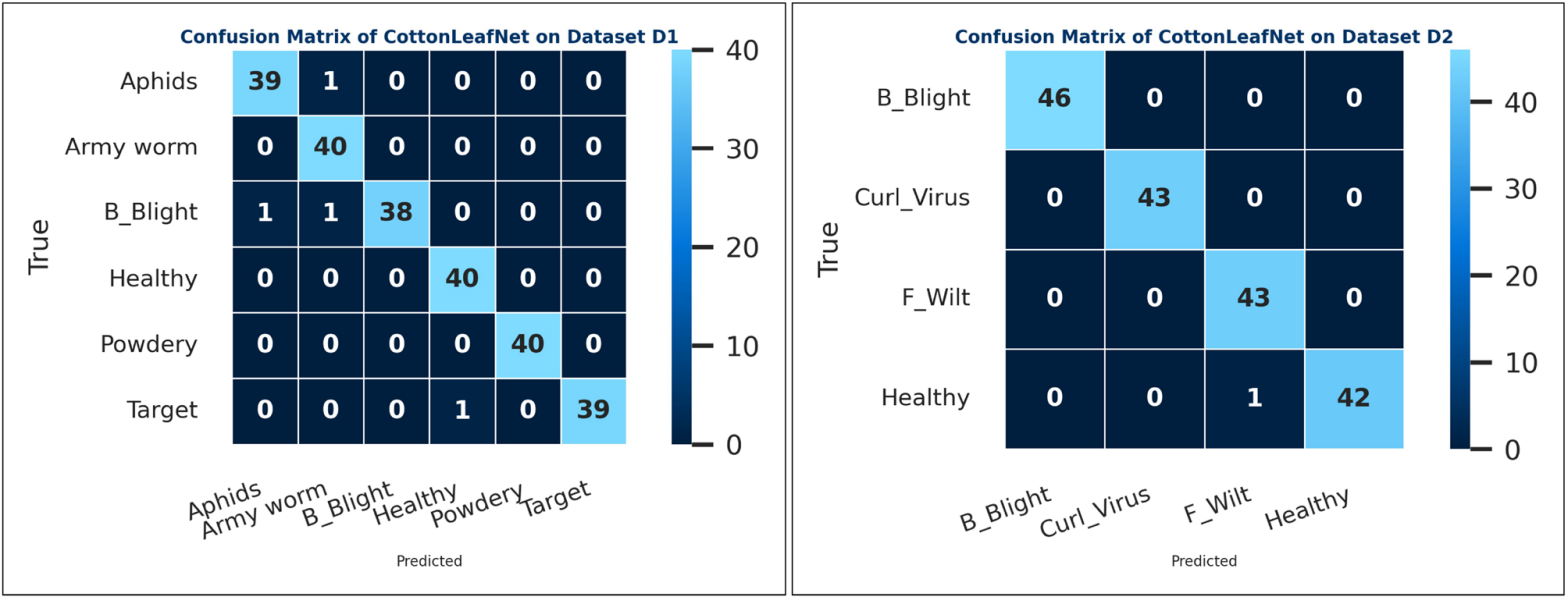
Confusion matrices for the proposed CottonLeafNet. Left: Dataset D1. Right: Dataset D2. The matrices illustrate class-level prediction performance and correct versus misclassified instances across all disease categories.

The confusion matrices in Fig. 7 illustrate the class-level prediction outcomes of the proposed *CottonLeafNet* across both datasets, providing deeper insight into its per-class reliability and generalization. In Dataset D1, the model achieves a very similar classification performance in all six categories. In particular, everything that was identified as *Healthy*, *Powdery Mildew* and *Army worm* was identified correctly, but only slight misclassification was reported. One of the images of the *Aphids* was falsely identified as an image of an *Army worm*, and two images of *Bacterial Blight* were falsely identified as an image of an *Aphid* and an image of an *Army worm*, respectively. There was also one incorrect classification of one of the samples as a *Target Spot* as *Healthy*. Even with such few mistakes, this general trend of the prediction points to the strong ability of the model to differentiate leaf patterns that are morphologically similar. The few misclassifications mostly involve the difference between *Aphids* and *Bacterial Blight*, two states that have nuanced similarities in terms of infection texture such as yellow spots and slight vein malformations whose evidence is sometimes confused during the initial disease phases.

In Dataset D2, the recognition of *CottonLeafNet* is close to perfect in all four categories. All of the samples of the *Bacterial Blight*, *Curl Virus*, and *Fusarium Wilt* were properly identified and the only misidentified sample as the *Bacterial Blight* was identified as *Healthy*. This slight confusion is perhaps due to the similarity of visual symptoms of early bacterial infection and non-infectious discoloration patterns of healthy leaves. In general, the results of the confusion matrices validate the idea that the proposed model successfully reduces inter-class confusion and maintains the stable accuracy of the models across datasets with varying disease compositions. Here, we also added some misclassified samples from both datasets to further evaluate model performance.

**Fig. 8:**
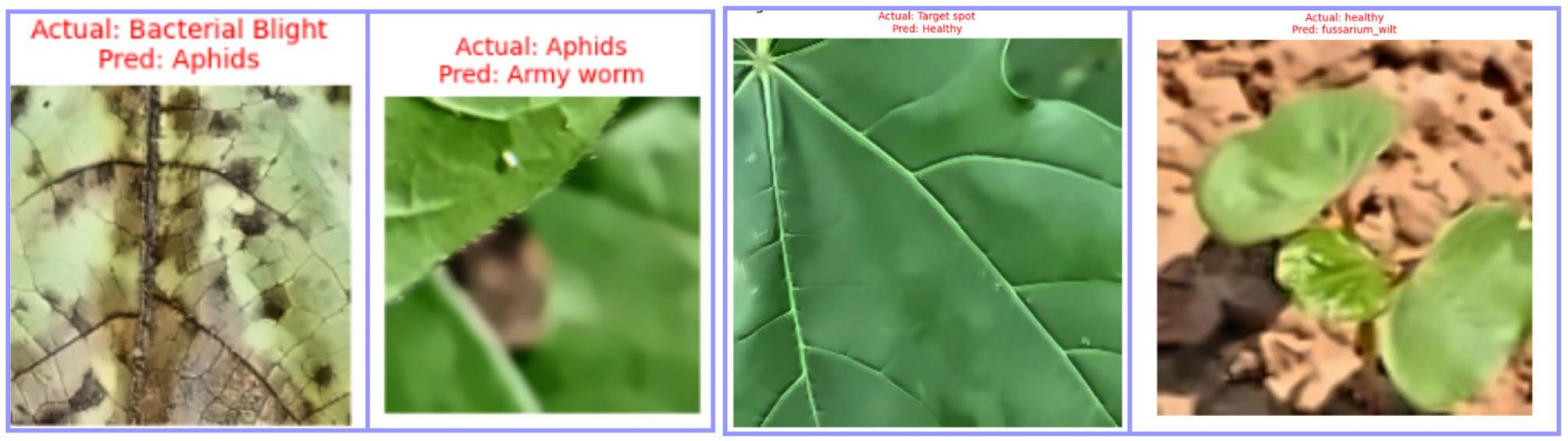
Some Misclassified images showing actual versus predicted labels from both datasets.

The Fig. 8 illustrates examples of misclassified cotton leaf images from both datasets, highlighting cases where the model predicted an incorrect class. It helps to visually analyze the types of errors, such as confusion between visually similar disease symptoms or early-stage infections that resemble healthy leaves, providing insights into the model’s limitations and areas for further improvement.

### Computational cost analysis of all evaluated model

Table 8 offers an overall overview of the computational costs of each of the considered models on Dataset 1 and Dataset 2, such as training time, inference time, per sample inference, gpu memory utilization and RAM usage. ShuffleNetV2 and MobileNetV2 are lightweight architectures with the quickest training and inference times (from 0.0016 to 0.0017 s per sample) across both datasets with a memory footprint of less than 400MB on the GPU. SqueezeNet proves to be efficient in competitive inference with moderate memory needs, and therefore is applicable to resource constrained deployments. Furthermore, further networks like VGG19 and ResNet101 have significantly more expensive training times (up to 470 s on Dataset 1) and larger memory allocations on the GPU (up to 4.77 GB on Dataset 2), which is the computational tradeoff of a more deeply trained network. EfficientNet B0 will bring a moderate trade-off, with a medium training time and memory consumption, and good predictive accuracy. The proposed CottonLeafNet has the largest computational requirement and the training times of 994.87 s and 408.47 s on Dataset 1 and Dataset 2, respectively, and the memory required by the GPUs is 911.80 MB and 2.41 GB. Although these training costs are high, per sample inference is low (0.51 to 0.40 s), which is practical to be applicable in real time or near real time forecasts. In line with this, despite the fact that, on the one hand, the training of the *CottonLeafNet* is more resource consuming, on the other hand, it provides an efficient trade off between the training and inference of the network by providing high accuracy and robustness.

**Table 8:** Computational Cost Comparison of Models on Dataset 1 and Dataset 2.

**Model**	**Dataset 1**					**Dataset 2**				
	**Total (s)**	**Inf. (s)**	**Per Sample (s)**	**GPU Mem (MB)**	**RAM (MB)**	**Total (s)**	**Inf. (s)**	**Per Sample (s)**	**GPU Mem (MB)**	**RAM (MB)**
ShuffleNetV2	136.17	0.40	0.0017	94.55	2136.94	281.60	0.37	0.002132	93.94	1344.57
SqueezeNet	172.14	0.36	0.001519	266	2380	138.89	0.29	0.001630	266	2110
VGG19	470.22	0.69	0.002866	1470	2380	217.07	0.57	0.003276	4770	2110
ResNet101	294.19	0.66	0.002730	445.96	1461.29	213.12	0.51	0.002907	444.97	1473.11
MobileNetV2	143.30	0.37	0.001556	368.71	1463.29	159.51	0.29	0.001672	366.94	1474.61
EfficientNet-B0	355.21	0.41	0.001715	375.10	1467.91	144.33	0.32	0.001817	375.36	1479.23
CottonLeafNet	994.87	0.51	0.00213	911.80	2709.98	408.47	0.40	0.00229	2412.45	2401.20

### Performance evaluation and reliability metrics

The results on the performance evaluation and reliability metrics of all models on datasets D1 and D2 are reported in Table 9. To evaluate the generalization ability of the individual models and how much they are consistent in predictive accuracy on different datasets, the metrics have been evaluated independently on D1 and D2.

In the case of Dataset D1, *CottonLeafNet* achieves the highest Cohens Kappa at 0.9800, the lowest Brier score at 0.0051, and the tightest confidence interval of the accuracy at [0.9667, 0.9958], which demonstrates that it is highly consistent with the actual labels as well as the well-calibrated predictions. Its mean PV (PPV) of 0.9838 and a mean NPV of 0.9967 are also the most favorable of the tested models which depict the consistency of reliable prediction of both positive and negative classes.

On Dataset D2, *CottonLeafNet* achieves the largest Cohens Kappa (0.9924) and NPV (0.9981), but the Brier score and the average PPV of *CottonLeafNet* is slightly lower compared to those of EfficientNetB0 and ShuffleNetV2 respectively. However, *CottonLeafNet* is still the most balanced and robust in terms of overall performance, which is demonstrated by its thin accuracy confidence interval of [0.9829, 1.0000] and competitive reliability indicators against all evaluation criteria. This result proves that the ensemble approach effectively combines the capabilities of its backbone architectures to attain better generalization on the two datasets.

**Table 9:** Performance Evaluation and Reliability Metrics for Dataset D1 and D2.

**Dataset**	**Model**	**Cohen’s**	**Brier**	**Accuracy 95% CI**	**Mean**	**Mean**
		**Kappa**	**Score**	**[Lower, Upper]**	**PPV**	**NPV**
**D1**	ShuffleNetV2	0.9550	0.0097	[0.9375, 0.9833]	0.9636	0.9926
	SqueezeNet	0.9600	0.0079	[0.9417, 0.9875]	0.9668	0.9933
	VGG19	0.9450	0.0103	[0.9250, 0.9792]	0.9548	0.9909
	ResNet101	0.9500	0.0099	[0.9333, 0.9833]	0.9608	0.9917
	MobileNetV2	0.9550	0.0098	[0.9375, 0.9833]	0.9643	0.9925
	EfficientNet-B0	0.9700	0.0082	[0.9542, 0.9918]	0.9760	0.9950
	**CottonLeafNet**	**0.9800**	**0.0051**	**[0.9667, 0.9958]**	**0.9838**	**0.9967**
**D2**	ShuffleNetV2	0.9848	0.0059	[0.9593, 0.9969]	0.9889	0.9962
	SqueezeNet	0.9771	0.0073	[0.9600, 1.0000]	0.9838	0.9944
	VGG19	0.9619	0.0104	[0.9429, 0.9943]	0.9724	0.9907
	ResNet101	0.9771	0.0075	[0.9600, 1.0000]	0.9833	0.9944
	MobileNetV2	0.9848	0.0046	[0.9714, 1.0000]	0.9885	0.9962
	EfficientNet-B0	0.9848	0.0041	[0.9714, 1.0000]	0.9885	0.9962
	**CottonLeafNet**	**0.9924**	**0.0044**	**[0.9829, 1.0000]**	0.9943	**0.9981**

### Unified model assessment across combined (D1+D2) datasets

In order to test the real-world application of the suggested CottonLeafNet, two datasets D1 and D2, in which the model had achieved the best results so far, were merged into one dataset that included all eight distinct cotton leaf disease types. Each of the models was re-trained using this combined data to evaluate their capabilities to work with diverse and heterogeneous data. The results of all the MHSA enhanced CNN models in terms of test performance on the unified dataset are summarized in Table 10. The findings suggest that CottonLeafNet outperforms all the baseline models with a precision of 0.9908, a recall of 0.9904, F1-score of 0.9904 and an accuracy of 0.9904. This indicates that the model is able to achieve consistency throughout all disease classes and be able to accommodate changes in leaf appearance and disease symptoms. Also, the ResNet101 and EfficientNet-B0 are some of the best baseline models with an accuracy of above 0.98, demonstrating the benefits of increased depth to extract intricate hierarchical leaf appearances. ShuffleNetV2 and SqueezeNet are also lightweight networks that provide competitive performance. The overall excellence of CottonLeafNet on both D1 and D2, as well as the combined dataset, shows that it is highly robust, generalizable and applicable to the real-world cotton leaf detection of the disease. These findings confirm the practical value of CottonLeafNet for real-world cotton leaf disease detection. We also presents the confusion matrix of the proposed model on the merged dataset below to visually present how well the model works to distinguish among all the eight classes of diseases.

**Table 10:** Test Performance of All MHSA-Enhanced CNN Models on Combined D1 and D2 Datasets.

**Model**	**Precision**	**Recall**	**F1-score**	**Accuracy**
ShuffleNetV2	0.9704	0.9687	0.9689	0.9687
SqueezeNet	0.9671	0.9663	0.9663	0.9663
VGG19	0.9660	0.9639	0.9641	0.9639
ResNet101	0.9811	0.9807	0.9807	0.9807
MobileNetV2	0.9745	0.9735	0.9737	0.9735
EfficientNet-B0	0.9809	0.9807	0.9806	0.9807
Proposed CottonLeafNet	0.9908	0.9904	0.9904	0.9904

**Fig. 9:**
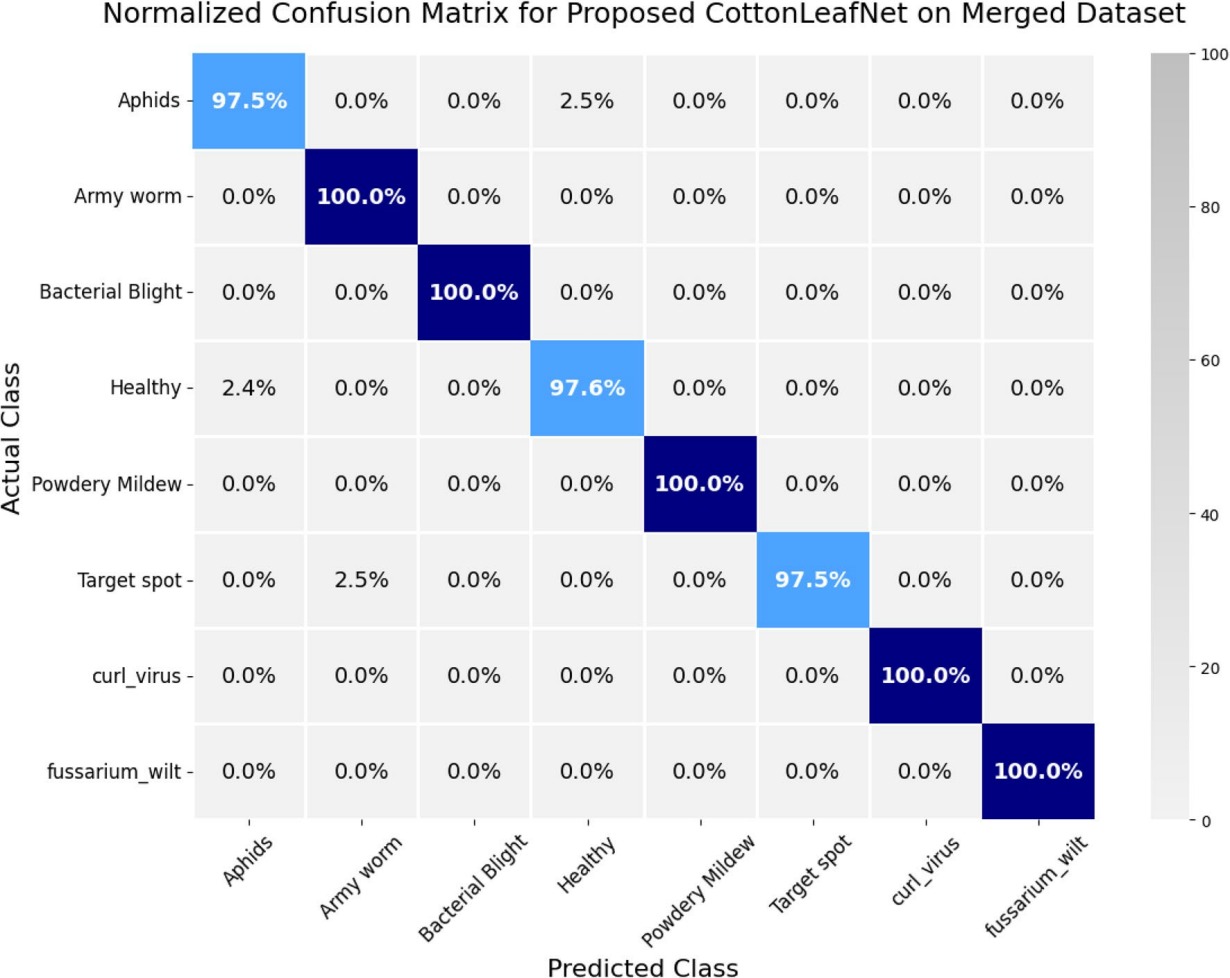
Normalized Confusion matrix of CottonLeafNet on the merged dataset of eight cotton leaf disease classes. Most classes are correctly identified, with only minor misclassifications in Aphids, Healthy, and Target spot.

The normalized confusion matrix of CottonLeafNet on the merged dataset containing eight cotton leaf disease classes are shown in Fig. 9. The model has a perfect classification rate of 100% in Army worm, Bacterial Blight, Powdery Mildew, curl virus and fusarium wilt with no misclassification cases in these classes observed. The Aphids, Healthy and Target spot classes have minor misclassifications. In particular, 2.5% of Aphids samples have been misclassified on the Healthy, 2.4% Healthy samples have been mistaken with Aphids and 2.5% Target spot samples have been mistaken with Army worm. In general, the confusion matrix shows that CottonLeafNet is a reliable disease classifier across the disease categories, which implies good performance and feasible possibilities of automated cotton leaf disease identification.

### Ablation study: contribution of MHSA to model performance

Table 11 also provides the test accuracy of various CNN models without MHSA as well as the percentage decrease relative to MHSA based models in Table 4. In Dataset D1, the most significant performance decreases are found in ShuffleNetV2 and VGG19 (5.42% each), which means that MHSA significantly improves feature representation in lightweight networks. Conversely, CottonLeafNet has a low decrease of 0.41%. In Dataset D2, the deletion of MHSA results in a 12.0% drop in ShuffleNetV2, explaining why it is an important factor in enhancing generalization between datasets with differing disease categories. In addition, CottonLeafNet experiences a decline of 1.72%. Also, across both Dataset D1 and D2, removal of MHSA causes notable performance drops in other models as well. Overall, MHSA consistently enhances accuracy by refining disease relevant features and improving discriminative performance, particularly in smaller and moderately deep networks.

**Table 11:** Test Accuracy of Different Models on Cotton Leaf Datasets without MHSA and Performance Decrease (%) compared to MHSA-enabled models.

**Model**	**D1 Test Accuracy**	**D2 Test Accuracy**	**D1 Decrease (%)**	**D2 Decrease (%)**
ShuffleNetV2	0.9083	0.8686	5.42	12.00
SqueezeNet	0.9583	0.9600	0.84	2.29
VGG19	0.9000	0.9371	5.42	3.43
ResNet101	0.9667	0.9714	−0.84	1.15
MobileNetV2	0.9292	0.9771	3.33	1.15
EfficientNet-B0	0.9667	0.9771	0.83	1.15
CottonLeafNet	0.9792	0.9771	0.41	1.72

### Cross-dataset evaluation of proposed CottonLeafNet

To examine the generalization behavior of the proposed CottonLeafNet, cross-dataset experiments were conducted using three independent datasets (D1, D2, and D3). The evaluation protocol included training the model on D1 and testing on D2, training on D2 and testing on D1, and training on the combined dataset (D1+D2) followed by testing on the external dataset D3.

Since datasets D1 and D2 share only two common disease classes, cross-dataset evaluation between these datasets was restricted to the overlapping classes to maintain comparability. As summarized in Table 12, CottonLeafNet achieved a test accuracy of 0.8717 when trained on D1 and tested on D2, and 0.9337 when the training and testing roles were reversed.

For external evaluation, the merged dataset D1+D2 was used for training, while D3 was reserved exclusively for testing. Dataset D3 includes six classes that overlap with those in D1+D2. To minimize potential data leakage, perceptual hashing^38^ was applied to identify duplicate images between the training and testing sets. Of the original 6,628 images in D3, 2,675 duplicates were removed, resulting in a test set containing only unique samples.

Under this configuration, CottonLeafNet achieved a test accuracy of 0.8305 on D3. Relative to within-dataset evaluations, the observed reduction reflects differences between datasets, including variations in image characteristics and class distributions. Overall, the results indicate moderate generalization performance when the model is applied to data from an external source.

**Table 12:** Cross-dataset evaluation of the proposed CottonLeafNet.

**Train**	**Test**	**Test Accuracy**
D1	D2	0.8717
D2	D1	0.9337
D1 + D2	D3	0.8305

### Proposed CottonLeafNet interpretability via Grad-CAM visualization

**Fig. 10:**
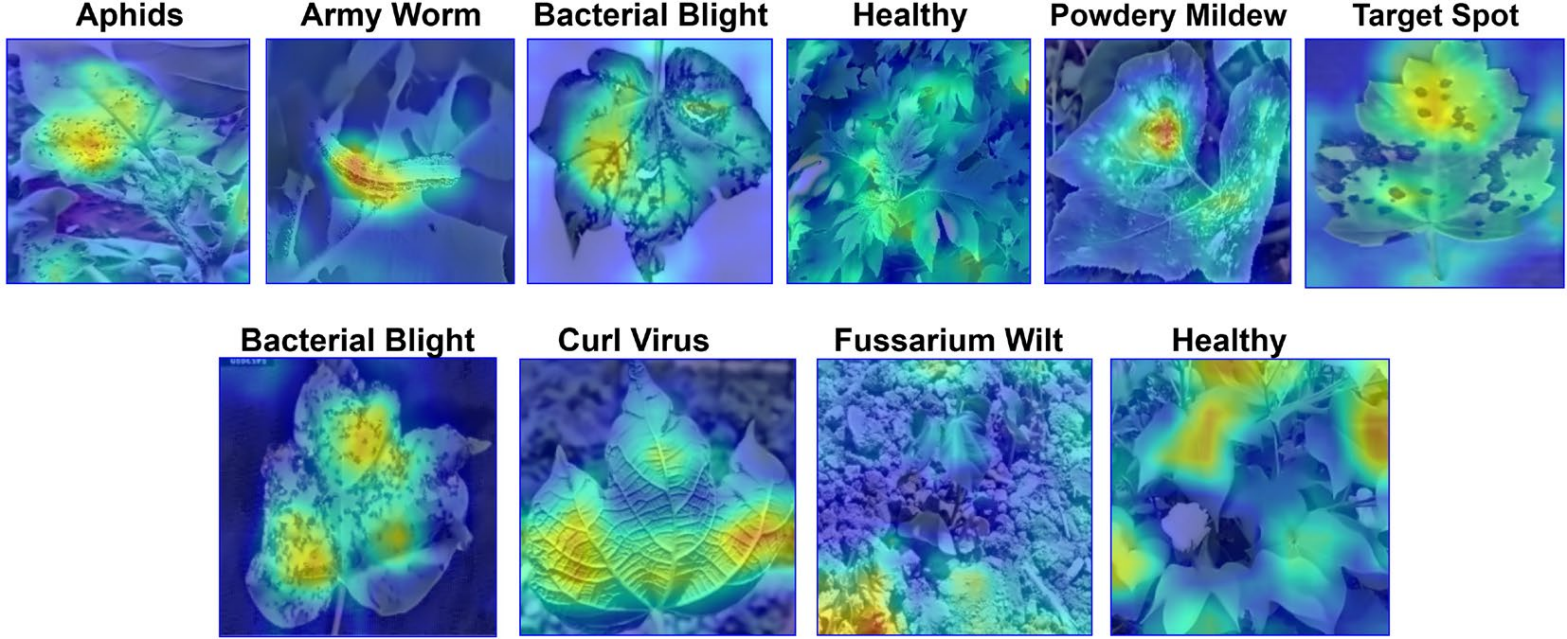
Grad-CAM visualizations for the proposed CottonLeafNet. Top row: sample images from Dataset D1. Bottom row: sample images from Dataset D2. The heatmaps highlight regions that contribute most to the predicted disease class.

To gain further insight into the decision making process of our proposed *CottonLeafNet*, Grad-CAM was used to visualize discriminative regions in cotton leaf image that affect a class prediction. As shown in Fig. 10, the model consistently focuses on disease relevant parts including lesions, spots, and affected leaf margins. The visualizations demonstrate that the model is attending to relevant features and not irrelevant background regions. The attention mechanism and MHSA modules are effective at capturing important patterns for classification. Manual inspection and confirmation by domain experts indicate that these regions of interest indeed correspond to actual disease symptoms. The model exhibits high quality in providing interpretable predictions while achieving high classification accuracy. The Grad-CAM interpretation highlights the reliability and applicability of the proposed approach for cotton leaf disease diagnosis.

### Interactive web application for automated cotton leaf disease diagnosis

**Fig. 11:**
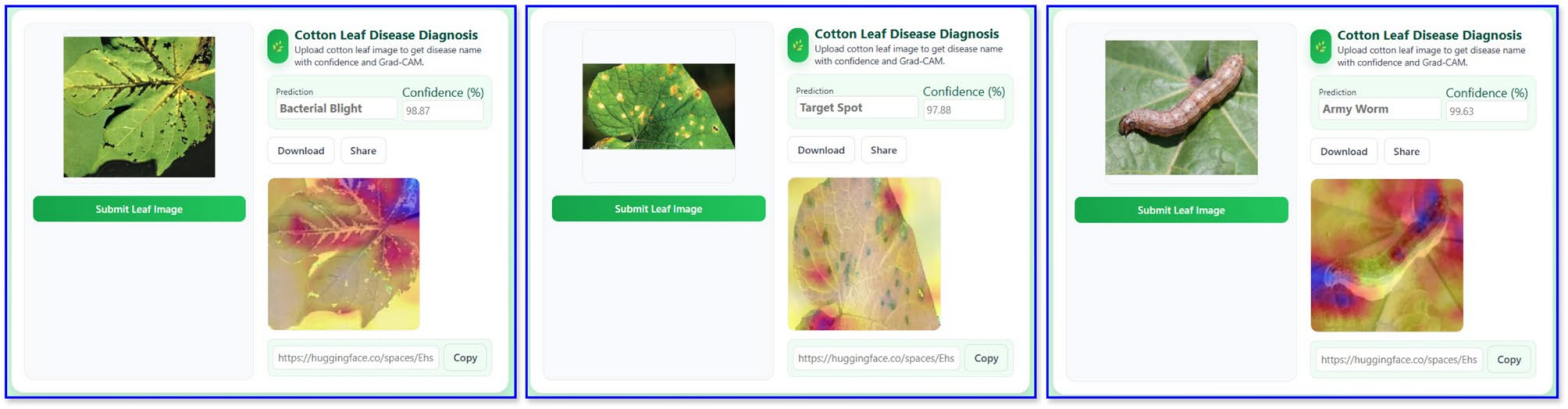
Interactive web application interface for automated cotton leaf disease diagnosis showing leaf image upload, disease prediction, confidence score, and Grad-CAM visualization.

An interactive web application was developed to show the practical potential of the proposed *CottonLeafNet*. The developed interface will allow users to input a cotton leaf image and immediately receive the predicted disease class, confidence score, and its Grad-CAM visualization focusing on the parts of the leaf that affected prediction. The model was deployed on Hugging Face using Gradio Spaces and is available at: https://huggingface.co/spaces/Ehsanul75/cottonleafdiseasediagnosis. A snapshot of the web application is given in Fig. 11. In that, the left panel depicts one leaf correctly identified class Bacterial Blight with confidence of 98.87%, the middle panel highlights *Target Spot* with a maximum confidence of 97.88%, and the rightmost panel shows correctly classified Army Worms with a confidence score of 99.63%. Most importantly, the web app provides predictions in around 1 second. This web platform is a user-friendly tool for disease diagnosis in real time, and it can be used for both research validation and practical applications in agricultural disease identification.

**Table 13:** Summary of previous works on cotton leaf disease detection.

**Study**	**Dataset Used**	**Model**	**Accuracy (%)**	**XAI**	**Web App**	**Limitations/Remarks**
^24^	1 (Self-collected)	ViT	96.72 (binary), 93.39 (multi-class)	No	No	Lacks explainable AI, cross-validation, computational cost analysis, and practical usability; evaluates only a single model.
^25^	1 (PlantVillage)	BERT-ResNet-PSO	98.5	No	No	No use of cross-validation, explainable AI, or computational cost analysis. Although 4,000 images are reported, the confusion matrix presents all 4,000 at once, raising concerns about result validity.
^19^	1 (Kaggle)	Xception	98.70	No	No	No explainable AI, cross-validation, or computational cost analysis; lacks usability testing and risk of overfitting observed in performance curves.
^26^	1 (Kaggle)	CNN	98.7	No	No	Lacks explainability and generalization analysis; omits computational cost evaluation and integration into real-world agricultural systems.
^20^	2 (Self-collected + Kaggle)	CNN	99.0	No	No	No use of explainable AI, cross-validation, or computational cost analysis; generalizability and practical deployment not discussed.
**Our Study**	3 (Kaggle D1, D2, Merged)	**CottonLeafNet**	98.33 (D1), 99.43 (D2), 99.08 (Merged)	Yes	Yes	**Limitations:** None reported. **Strengths:** Uses three benchmark datasets, employs explainable AI (XAI), applies cross-validation, cross dataset evaluation, analyzes computational cost, performs rigorous evaluation, deploys a user-accessible web app, and achieves negligible inference cost for real-time diagnosis.

### Comparison of the proposed framework with existing literature

Table 13 presents the literature summary of the past research on cotton leaf disease detection and compares it our proposed CottonLeafNet. Most available literature use single datasets and are based on a single model architecture like ViT, CNN, or Xception, attaining the accuracy of between 96.7 and 99.0 %. Nonetheless, such studies tend to lack explainable AI, cross validation, analysis of computational cost and practical considerations on usability, which restricts their reliability and interpretability as well as usability in the real world.

Conversely, the proposed CottonLeafNet relies on two benchmark datasets, employs explainable AI, utilizes five-fold cross validation, evaluates the computational cost, and is thoroughly evaluated. It is also implemented as a web application accessible to users with a small per sample inference time to be used in practice in real-time. This proves that CottonLeafNet not only achieves higher accuracy of 98.33% and 99.43% in D1 and D2 respectively but also addresses key gaps in interpretability, robustness, and usability that had not been witnessed in the past. So in summary, Our work is novel in the sense that we proposed an ensemble deep learning model, CottonLeafNet, that combines XAI, web app deployment, rigorous cross-validation, and the lower computational cost and generalizes effectively across three benchmark datasets. This ensures high robustness, interpretability and real-time usability of our proposed framework.

## Conclusion

This work presented *CottonLeafNet*, an attention-enhanced ensemble framework that integrates lightweight CNN backbones with MHSA modules to achieve accurate and computationally efficient cotton leaf disease classification. Through extensive preprocessing, augmentation, and five-fold cross-validation, the proposed model demonstrated strong robustness across multiple datasets. On D1 and D2, CottonLeafNet achieved test accuracies of 98.33% and 99.43%, macro F1-scores of 0.9833 and 0.9924, Cohen’s Kappa of 0.9800 and 0.9924, mean PPV of 0.9838 and 0.9943, and NPV of 0.9967 and 0.9981, respectively. Grad-CAM visualizations further confirmed that the model consistently attends to disease-relevant regions, strengthening interpretability and trustworthiness. The framework also offers practical value, achieving low inference times (0.51 s and 0.40 s per image) and supporting a real-time web application delivering predictions within approximately 1 s. Additionally, the unified eight-class dataset constructed from D1 and D2 yielded a test accuracy of 99.08%, which suggests that the model is able to handle more complex multiclass disease scenarios. Cross-dataset evaluation further shows that CottonLeafNet maintains moderate performance when trained and tested on different datasets, which provides an indication of its generalization capability.

Future work will focus on expanding dataset diversity, improving robustness against visually overlapping disease symptoms, and enhancing the web application for large-scale deployment, including mobile-friendly interfaces and cloud-supported inference. These efforts aim to facilitate timely, reliable, and interpretable disease diagnosis in real-world agricultural environments.

## Data Availability

The datasets analyzed during the current study are publicly available in the Kaggle repository. Dataset D1 can be accessed at  https://www.kaggle.com/datasets/ataher/cotton-leaf-disease-dataset/data, and Dataset D2 can be accessed at https://www.kaggle.com/datasets/seroshkarim/cotton-leaf-disease-dataset. The dataset used for cross-dataset testing is publicly available at: https://www.kaggle.com/datasets/saeedazfar/customized-cotton-disease-dataset%3fselect%3dCustomized+Cotton+Dataset-Complete.
